# Minimally invasive hybrid retrieval of a fractured sion guidewire during transcatheter coronary intervention: a case report of multidisciplinary collaborative management

**DOI:** 10.3389/fcvm.2026.1846838

**Published:** 2026-09-01

**Authors:** Xianliang Dai, Jiming Han, Yijing Guo, Gang Zhao, Chengxing Shen

**Affiliations:** Department of Cardiology, Shanghai Sixth People’s Hospital Affiliated to Shanghai Jiao Tong University School of Medicine, Shanghai, China

**Keywords:** brachial arteriotomy, complication management, coronary artery disease, guidewire fracture, multidisciplinary team collaboration, percutaneous coronary intervention

## Abstract

**Background:**

Guidewire fracture during percutaneous coronary intervention (PCI) is rare but serious, with management dependent on fracture characteristics. We report an innovative minimally invasive hybrid approach for this complication.

**Case summary:**

A 59-year-old male with diabetes/hypertension developed Sion guidewire fracture during LAD PCI. Percutaneous retrieval failed; conservative therapy was initially given. A minimally invasive hybrid “trans-brachial arteriotomy with endoluminal traction” retrieved the fragment 34 days post-fracture, with uneventful recovery.

**Discussion:**

Guidewire fracture occurs in 0.2%–0.8% of PCI cases. Histopathological evidence suggests that at 34 days, incomplete endothelialization facilitates rather than hinders retrieval. Although no standard retrieval window exists, delayed elective retrieval can achieve high success rates in stable patients. Traditional open surgery is traumatic; our hybrid approach avoids sternotomy, offering a novel salvage strategy valuable for interventional cardiologists and surgeons.

**Take home messages:**

Minimally invasive hybrid retrieval is safe and effective for PCI guidewire fractures refractory to percutaneous methods, emphasizing the value of multidisciplinary collaboration.

## Introduction

Percutaneous coronary intervention (PCI) is a primary revascularization strategy for coronary artery disease. Guidewire fracture is a rare [0.2%–0.8% of cases (1, 2)] yet challenging complication, with fragments posing risks of thrombosis, perforation, or embolism (3). Percutaneous retrieval is the first-line approach, but failed cases may require open surgery—an option that is traumatic and suboptimal. Herein, we report the successful use of an innovative minimally invasive hybrid approach for managing a complex Sion guidewire fracture, highlighting the critical role of multidisciplinary collaboration.

## Case report

### Patient information and medical history

A 59-year-old male with type 2 diabetes mellitus and hypertension was admitted on November 18, 2025, presenting with recurrent exertional chest tightness (duration >1 year) and left upper arm pain (duration 1 week). Initial coronary angiography performed on August 19, 2025, revealed severe multi-vessel disease: 90% stenosis in the proximal-to-mid left anterior descending artery (LAD) with mid-segment subtotal occlusion and TIMI 1 distal flow, 80%–90% stenosis in the distal left circumflex artery (LCX), and 90% stenosis in the proximal-to-mid right coronary artery (RCA). The patient and his family declined coronary artery bypass grafting (CABG) and opted for PCI, during which two drug-eluting stents were successfully implanted in the RCA. Post-procedurally, he was maintained on dual antiplatelet therapy, statins, and antidiabetic medications.

### Current admission and pre-procedural assessment

One week prior to the current admission, the patient experienced exertional chest tightness radiating to the left upper arm, relieved by 1–2 min of rest, suggesting recurrent myocardial ischemia. Admission electrocardiogram showed no acute ischemic changes, and cardiac enzyme levels were within normal limits. Given the clear symptoms and previously documented significant residual lesions in the LAD and LCX, intervention on the non-RCA vessels was planned.

A second coronary angiography on November 19, 2025, confirmed patency of the RCA stents, 90% stenosis at the LAD-first diagonal branch (D1) bifurcation, and unchanged LCX disease. After re-discussing the risks and benefits of CABG versus PCI, the patient again opted for PCI.Two drug-eluting stents (Firebird 2.5 × 29 mm and XIENCE 3.0 × 33 mm) were implanted in a telescoping fashion from the mid to proximal LAD and deployed at 12 atm.

### Procedure and complication

**Figure 1:**
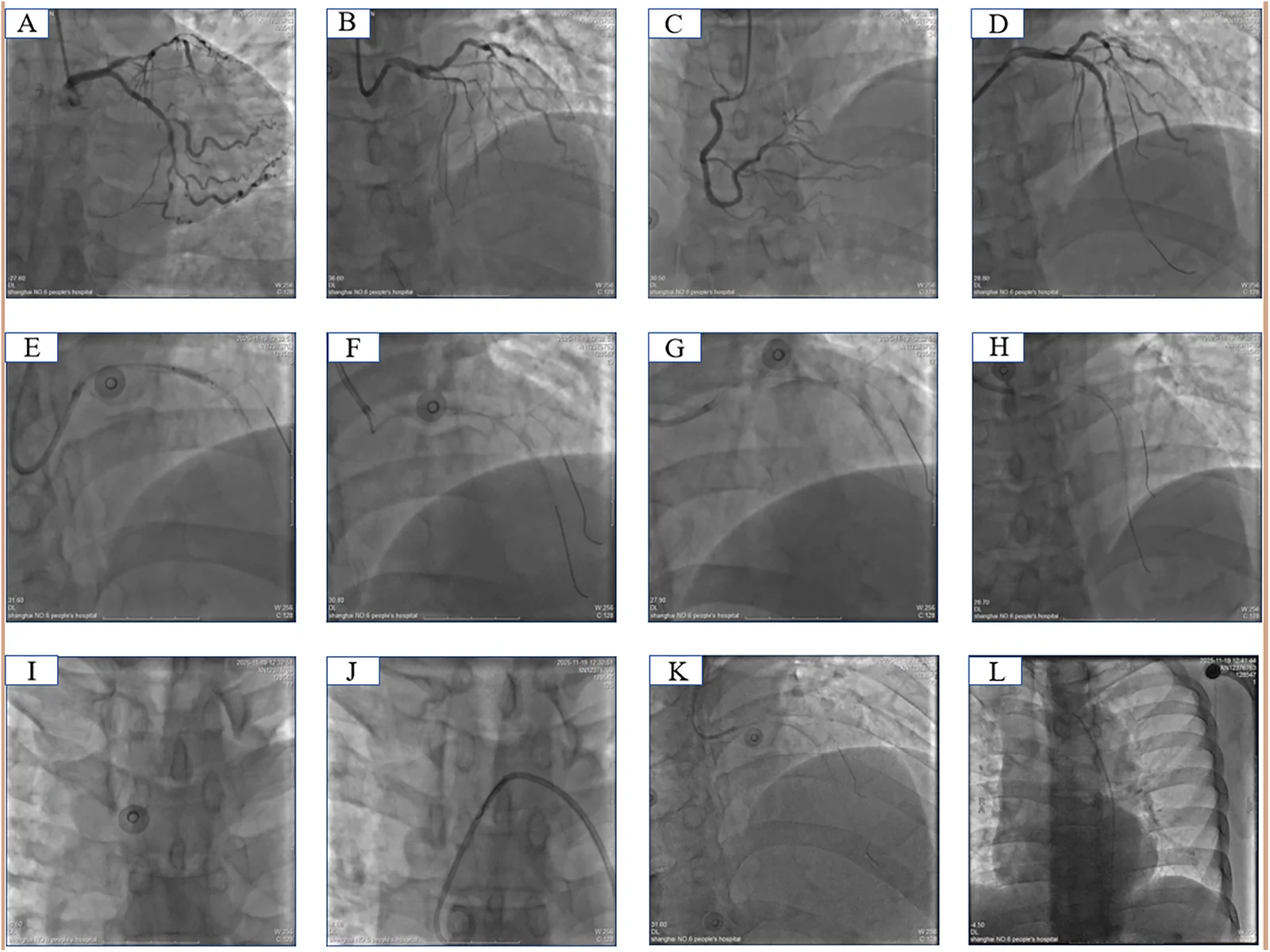
Guidewire Fracture developed during the second coronary angiography and interventional procedure, with the implementation of all available and attempted techniques for guidewire retrieval. **(A, B and C)** denote the respective angiographic findings of the left circumflex artery (LCX), left anterior descending artery (LAD) and right coronary artery (RCA). **(D**) denotes the follow-up angiographic findings of the left anterior descending artery (LAD) after stent implantation. **(E)** Balloon Dilation for Disentanglement: A 0.8 × 10 mm Niballon balloon was advanced to the D1 ostium and inflated to 6 atm in an attempt to release potential entrapment. **(F and G)** Microcatheter Advancement: Attempted to advance a microcatheter to ensnare or sheath the fragment, unsuccessful due to the D1 ostial angle and stent strut interference. **(H and I)** Shockwave Balloon for Plaque Modification: A GenNwave shockwave balloon was used to modify local calcified plaque, aiming to release the guidewire. **(J)** Snare Capture: Attempted using a vascular snare but failed to securely loop the slender fragment. **(K)** Compression Techniques: Attempted to “push” the fragment by compressing its proximal end within the guide catheter using an NC balloon and later a stent balloon. **(L)** Access Conversion: Due to radial artery spasm, a second access was established via the right femoral artery for further attempts, which also proved unsuccessful.

After implanting two stents in the proximal and middle segments of the LAD, we prepared to conclude the operation. When withdrawing the Sion guidewire, no resistance was initially encountered. However, despite significant withdrawal of the proximal guidewire, the distal end failed to exit. We subsequently performed fluoroscopic examination and found that the distal guidewire remained immobile at the distal D1. The operator then suspected guidewire fracture, but it was already too late. To maximize vascular integrity preservation, a sequential algorithm of percutaneous retrieval techniques was attempted. Initial low-pressure dilation of the first diagonal branch (D1) ostium was performed with a Niballoon 0.8 × 10 mm balloon for potential local entrapment relief, which was unsuccessful.we advanced the microcatheter along the guidewire toward the fractured fragment, but due to the dense obstruction of stent struts at the LAD/D1 bifurcation, anatomical angulation of the site, and severe calcification in this area, the microcatheter failed to cross the stent struts. Even after adjusting the angle of the guide catheter, the microcatheter still could not reach the fractured segment. Further fluoroscopic observation showed that the guidewire fragment was partially embedded in the vascular wall at the bifurcation with limited mobility. Given significant local calcification, plaque modification was conducted with a Gennwave shockwave balloon to facilitate guidewire liberation, with no discernible effect. Vascular snare deployment for fragment capture was then attempted but failed due to the fragment's high mobility and slender caliber. As a final percutaneous maneuver, compressive retrieval of the fractured guidewire stump was attempted via the guiding catheter with both an NC balloon and a Xinyue stent, without achieving displacement. Radial artery spasm developed, prompting conversion to a right femoral artery access; all percutaneous retrieval attempts were ultimately unsuccessful. Intraprocedural imaging confirmed TIMI grade 3 flow in the main left anterior descending coronary artery (LAD) (Figure 1).

An urgent intraprocedural multidisciplinary consultation was held with the interventional cardiology and cardiac surgery teams. Given the low acute risk of residual fragment-related complications (e.g., vascular perforation, embolization), procedural termination, conservative management, and close clinical surveillance were recommended. The procedure was subsequently aborted, and the patient was transferred back to the ward for continuous observation.

### Multidisciplinary consultation and initial decision

Post-procedurally, a multidisciplinary team (including cardiologists, cardiac surgeons, and interventional radiologists) recommended conservative management, noting that the small, flow-neutral guidewire fragment was likely to become endothelialized over time. Two external experts concurred, advising observation and intensified antiplatelet therapy. Conservative management—consisting of aspirin (100 mg/day) + clopidogrel (75 mg/day), statin therapy, and blood pressure/glucose control—was initiated, with close clinical follow-up planned.

### Decision pivot and hybrid surgical plan formulation

**Figure 2:**
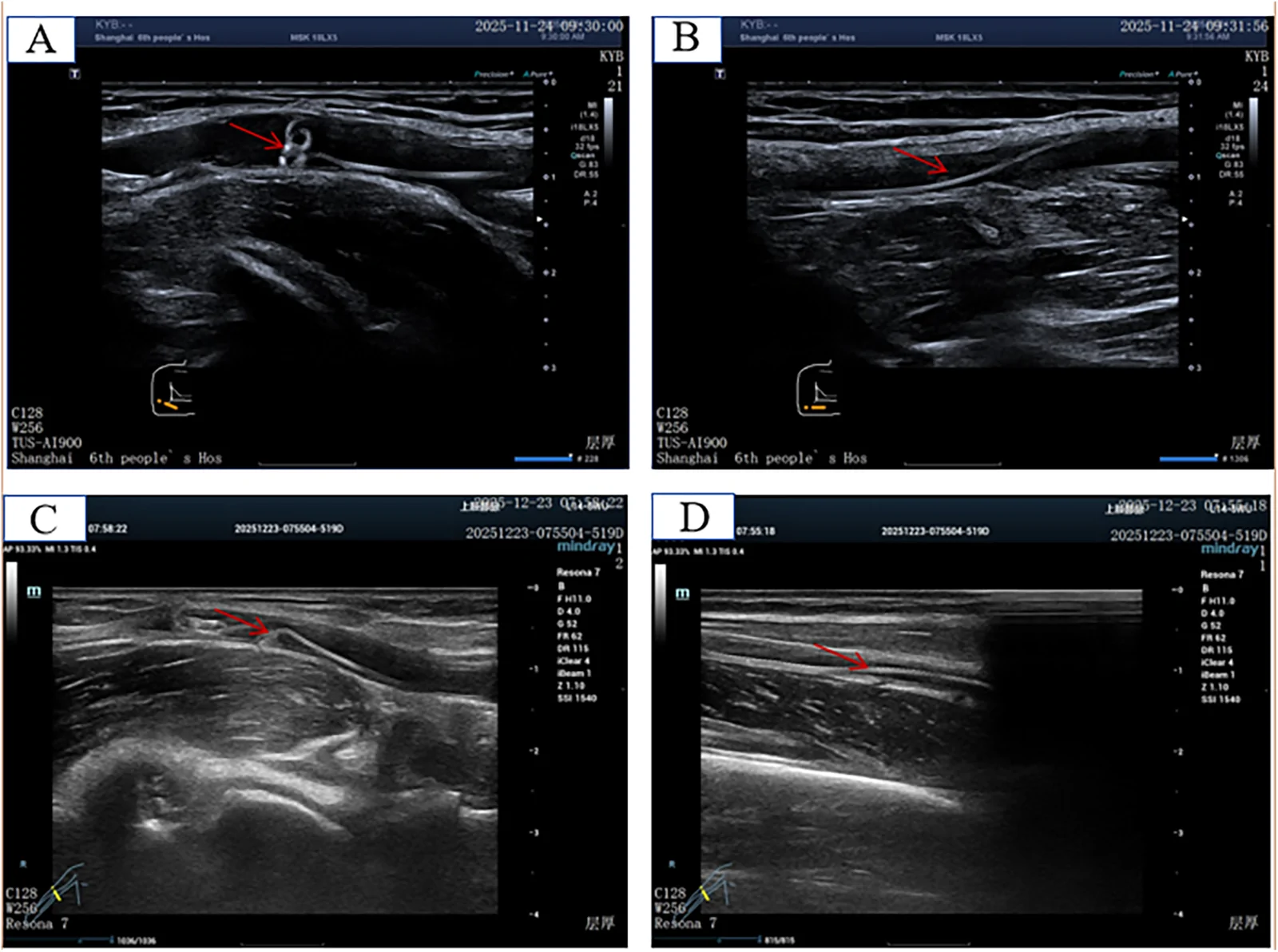
Guidewire fracture occurred in the D1 branch during the second interventional procedure, with results of upper extremity vascular ultrasonography performed on two subsequent occasions (24th November and 23rd December). The red arrow indicates the fractured guidewire stump (the stump is visible extending to the brachial artery).

Over the following weeks, the healthcare team engaged in multiple in-depth discussions with the patient. The patient expressed profound anxiety about the long-term presence of a foreign body, concern about potential late complications (e.g., thrombosis, device interference), and reported persistent, albeit improved, chest tightness. During this period, two vascular ultrasound examinations revealed that the distal end of the fractured guidewire fragment was located in the brachial artery (Figure 2). The team re-evaluated the situation, acknowledging that while short-term risks were low, the guidewire as a permanent foreign body at a critical bifurcation carried unknown ultra-long-term (10–20 years) risks and could potentially compromise future treatment options for that segment.

After renewed multidisciplinary discussion and with full respect for the patient's autonomy, the team decided on active intervention. To avoid the significant trauma of traditional open-heart surgery, the cardiac surgery and interventional cardiology teams collaboratively designed an innovative minimally invasive hybrid surgical plan: “Trans-brachial Arteriotomy Combined with Endoluminal Traction Retrieval.” This approach aimed to establish a controlled pathway through a small peripheral incision to safely exteriorize the coronary guidewire fragment.

### Hybrid procedure (December 23, 2025)

**Figure 3:**
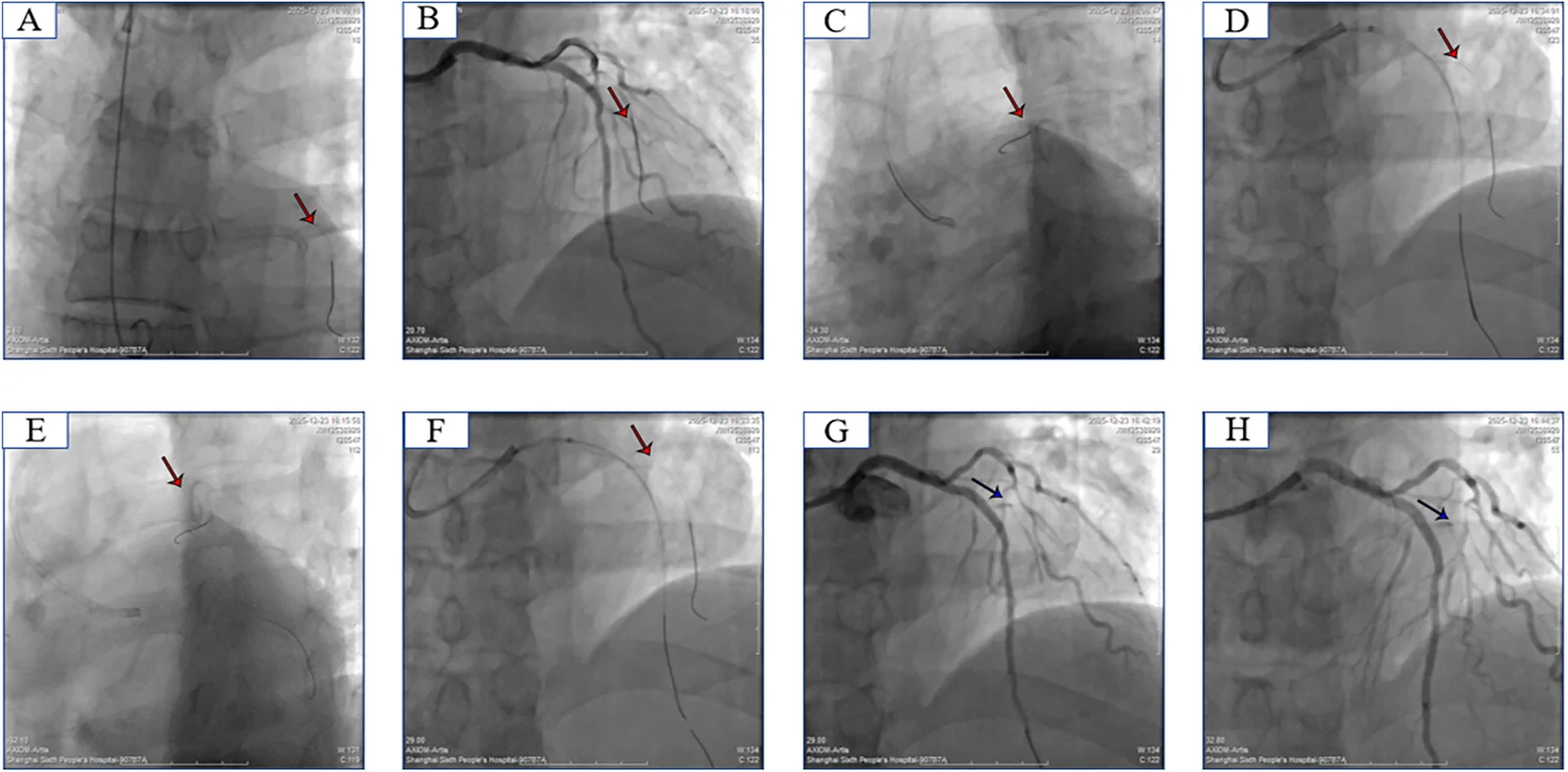
Retrieved fractured sion guidewire following minimally invasive hybrid trans-brachial arteriotomy with endoluminal traction. The fragment extended from the distal D1 branch to the right brachial artery at the cubital fossa, without evidence of perforation or significant endothelial adhesion.

The patient was positioned supine on a digital subtraction angiography (DSA) capable hybrid operating table. A 2 cm longitudinal incision was made over the right antecubital fossa. The brachial artery was dissected free. After placing proximal and distal vessel loops for control, a transverse arteriotomy was made on the anterior wall. Using a nerve hook, the proximal end of the Sion guidewire fragment within the brachial artery was carefully hooked and retrieved. An 8F vascular sheath was introduced retrograde over the retrieved guidewire end. A surgical suture was securely tied to this guidewire end. Subsequently, an 8F guide catheter (IL 3.5) was gently advanced over the suture to the left coronary ostium. A Runthrough guidewire was advanced through the guide catheter into the distal LAD for support. An extension catheter was then advanced over the suture and the *in-situ* guidewire remnant to the proximal edge of the LAD stent. For added system stability, a second Runthrough guidewire was placed in the distal LAD. Under fluoroscopic guidance, gentle and continuous traction was applied to the pre-attached distal suture, successfully extracting the entire guidewire fragment lodged at the LAD-D1 bifurcation. After confirming complete retrieval, post-dilation of the proximal LAD stent segment was performed using an NC balloon(3.0 × 15 mm). Final angiography showed excellent LAD patency (TIMI 3 flow) without dissection. Finally, the brachial arteriotomy was closed with a continuous 5–0 Prolene suture. Radial artery pulse was confirmed, and the subcutaneous tissue and skin were closed in layers (Figure 3).

### Postoperative course and follow-up

#### Hospital stay

The patient reported no further chest tightness or left arm pain. The brachial incision healed well without signs of infection, hematoma, or limb ischemia. Dual antiplatelet therapy, statins, antihypertensives, and antidiabetic medications were continued.

#### Follow-up plan

**Figure 4:**
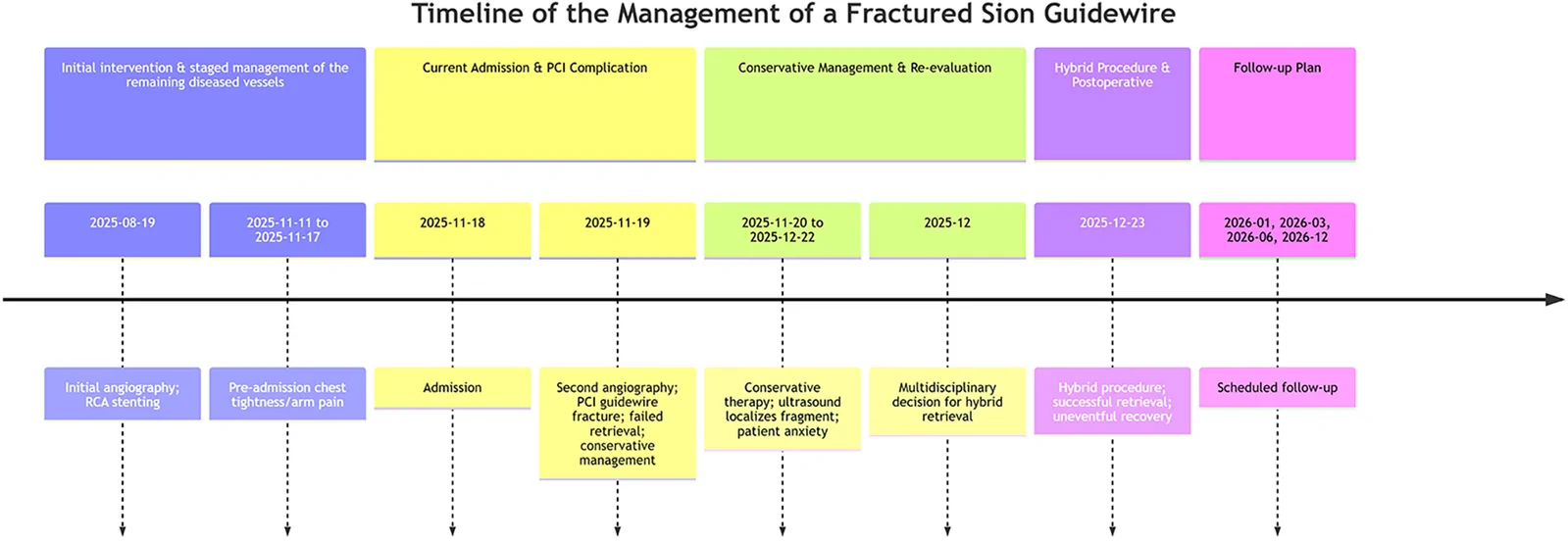
Timeline of the management of a fractured sion guidewire.

Outpatient follow-up was scheduled at 1, 3, 6, and 12 months. Assessments will include evaluation of anginal symptoms, electrocardiogram, echocardiography for cardiac function, and coronary CTA at 6–12 months to assess LAD/D1 patency and vascular healing at the retrieval site. The timeline of the management of the fractured Sion guidewire is shown in Figure 4.

## Discussion

This case comprehensively illustrates the stepped decision-making and therapeutic process for managing a complex PCI guidewire fracture complication. Its core value lies in demonstrating the successful application of an innovative hybrid procedure through multidisciplinary collaboration.

This fracture involved a Sion guidewire, a hydrophilic-coated wire commonly used in complex lesions. Potential causes include: (a) metal fatigue from repeated manipulation in a severely calcified, angulated bifurcation (LAD-D1); (b) mechanical entrapment by neointimal stent struts or existing plaque; (c) excessive rotation or traction upon encountering resistance during withdrawal. Preventive measures include: avoiding repetitive to-and-fro movement of the guidewire in complex lesions; immediately stopping and using angiography or IVUS to identify the cause if resistance is met during withdrawal, rather than forceful pulling; considering more durable dedicated guidewires for extremely calcified lesions (3).

The histomorphological study by Killer et al. (4) provides a pathological basis for understanding this process. They found that neointima formation begins as early as 5 days after foreign body retention, yet thrombus organization and endothelialization remain incomplete even within the observation period of up to 74 days. This suggests that at the 34-day delay window, endothelialization is still in an active phase, and the fragment has not been completely and firmly encapsulated, theoretically allowing for snare retrieval. Although there is no universally established “optimal retrieval time window,” the study by Önal et al. (5) demonstrated that the overall success rate of elective interventional retrieval can reach as high as 90%, even in non-emergent settings. This indicates that in the absence of urgent retrieval indications, a short period of clinical observation (e.g., the 34 days in this case) to optimize the procedural strategy is reasonable. The key decision-making factor should be whether the fragment has already caused symptoms, thrombosis, or risk of perforation, rather than relying solely on the duration of retention.

The decision-making process in this case is instructive. Initial multidisciplinary consensus favored conservatism, based on a rational assessment of “low acute risk,” aligning with guidelines and expert consensus for managing asymptomatic, non-flow-limiting small device fragments. However, the decision was not static. The patient reported persistent intrusive thoughts about the broken wire, difficulty sleeping, and fear of future complications despite repeated reassurance. This psychological burden became the primary factor tipping the risk–benefit balance toward intervention, even in the absence of clinical symptoms or imaging progression. Patient values and preferences (anxiety about the foreign body), re-evaluation of “ultra-long-term uncertainty,” and the feasibility of minimally invasive surgical techniques collectively drove the shift toward active intervention. This highlights the importance of shared decision-making models in modern individualized medicine.

The “trans-brachial arteriotomy with endoluminal traction” technique employed represents an innovative hybrid solution. Compared to thoracotomy, the brachial arteriotomy is less traumatic, promotes faster recovery, and avoids sternotomy-related complications. The entire traction process was performed under fluoroscopy, with the suture providing smooth guidance. Utilizing the native guidewire as a “rail” aligns traction force with the guidewire axis, reducing coronary injury risk (6).

As documented in the literature, percutaneous retrieval can be impeded by severe calcification or stent strut entrapment, necessitating surgical bailout. Koya et al. (7). demonstrated that a minimally invasive off-pump approach via small thoracotomy successfully retrieved an undeflatable balloon after failed catheter-based attempts. Similarly, Réza et al. (8). systematically analyzed the mechanisms of coronary guidewire and stent entrapment during PCI, underscoring the limitations of endoscopic retrieval tools such as snare or bioptome when encountering severe calcification or stent strut obstruction. Together, these reports illustrate the clinical value of hybrid techniques that combine minimal surgical access with endoluminal traction, supporting the uniqueness and clinical value of our approach.

The crucial aspect was establishing a stable, low-resistance traction pathway from the exterior to the intracoronary foreign body. Using an extension catheter to cover the intracoronary path and a second guidewire for added support were critical details ensuring successful retrieval and avoiding vascular injury.

The successful management of this case was highly dependent on seamless collaboration among interventional cardiologists, cardiovascular surgeons, imaging specialists, and nursing staff. Interventional cardiologists provided expertise in coronary anatomy and devices; cardiac surgeons contributed skills in vascular access, suturing, and managing potential hemorrhage; and joint preoperative planning ensured procedural fluency. Establishing an institutionalized multidisciplinary complication review mechanism is foundational for managing such complex, high-risk scenarios.

## Study limitations

This is a single-center case report. The generalizability and long-term outcomes of this hybrid technique require validation in larger studies. Furthermore, reporting a successful case may not encompass the experience with all types of guidewire fractures.

## Conclusion

For coronary guidewire fractures refractory to conventional percutaneous methods, they should not be hastily deemed irretrievable or necessarily mandate open-heart surgery. This case demonstrates that through deep collaboration between cardiology and cardiac surgery, innovative minimally invasive hybrid procedures can serve as a safe and effective salvage strategy. This approach successfully balances therapeutic efficacy with minimal invasiveness, representing a significant direction in managing complex interventional complications. Future efforts should focus on accumulating more cases to refine indications, optimize technical details, and establish standardized protocols.

## Data Availability

The original contributions presented in the study are included in the article/Supplementary Material, further inquiries can be directed to the corresponding author.
